# Clinical Profile, Risk Factors and Outcomes of Ectopic Pregnancy in a Tertiary Care Hospital: A Prospective Indian Study

**DOI:** 10.7759/cureus.49483

**Published:** 2023-11-27

**Authors:** Neelam Nalini, Kshitij A Singh, Neetu S, Ankita Kumari

**Affiliations:** 1 Obstetrics and Gynaecology, Rajendra Institute of Medical Sciences, Ranchi, IND; 2 Pediatrics, Jawaharlal Nehru Medical College, Datta Meghe Institute of Medical Sciences, Wardha, IND; 3 Ophthalmology, Maharaja Krishna Chandra Gajapati Medical College & Hospital, Brahmapur, IND

**Keywords:** methotrexate, ruptured, salpingectomy, tubal pregnancy, ectopic pregnancy

## Abstract

Objectives

To determine the clinical profile, management and outcomes in patients of ectopic pregnancy (EP) presenting to the hospital.

Methods

A prospective observational study was done on 75 women with ectopic pregnancy among a total of 1350 women who attended the Gynecology department over a period of 18 months from December 2020 until June 2022. The details of patients such as age, socioeconomic status, referral, symptoms, obstetric history, and signs and symptoms were recorded. Ultrasound was done and the site of ectopic pregnancy was determined. Management of patients was done according to hospital protocol; outcomes were recorded.

Results

The frequency of EP in the present study was 5.55%. The majority of the cases (60%) were between 20-30 years of age, from the lower middle class (57%), referral cases (63%), and multigravida (i.e. >G4) (31%) cases. Amenorrhea was the most common symptom seen in 73 (97.33%) cases. On ultrasound, the most common finding was tubo-ovarian mass (33.33%). In the majority of patients [28 (37.33%)], the ampullary region in the fallopian tube was the site of EP. History of pelvic inflammatory disease was most common risk factor [26 (19.5%)]. Surgical management was done in 74 cases and one case was managed medically. Salpingectomy alone was done in 47 (62.67%) cases. Rupture ectopic pregnancy was seen in 71 (95%) cases where all cases needed blood transfusion. One patient needed ventilatory support/ICU admission and had mortality.

Conclusion

We report a frequency of 5.55% for EP. The patients with EP were in the age groups of 20-40 years and belonged primarily to lower socioeconomic class. The most common symptom was amenorrhea. The most common site of ectopic pregnancy was the ampullary region. Rupture ectopic is a common worrisome complication. Early diagnosis of the site and surgical management is the key to better management of ruptured cases.

## Introduction

Ectopic pregnancy (EP) is a complication that occurs in the first trimester of pregnancy when an embryo implants outside of the uterus [1]. In India, the incidence has been reported in the range of 0.91-2.3% [1,2]. The most frequent risk factors of EP include a history of abortions and pelvic inflammatory disease (PID) [3,4]. Because the classic symptoms triad of amenorrhea, abdominal pain, and vaginal bleeding is present only in 30% to 40% of patients with EP, the diagnosis requires a high index of clinical suspicion [5].

The EP spectrum includes asymptomatic patients as well as ruptured ones that present in shock. Increased morbidity and occasionally even fatality are consequences of delayed diagnosis [6]. It could even have an impact on her future fertility if not treated instantly and effectively [7].

The present study was done with the aim to determine the clinical profile, risk factors, management and outcomes in patients of EP presenting to the hospital.

## Materials and methods

A prospective observational study was done on 75 women with ectopic pregnancy among a total of 1350 women who attended the Obstetrics and Gynaecology department over a period of 18 months from December 2020 until June 2022. Adult females who had ectopic pregnancy were included. Ectopic pregnancy was suspected on the basis of (1) clinical symptoms of missed periods, nausea, vomiting, vaginal bleeding, breast tenderness, frequent urination or pelvic pain, (2) imaging characteristics on ultrasound with no signs of pregnancy or the presence of an adnexal mass, or absence of an intrauterine gestational sac (transvaginal ultrasound) with the background of β-hCG levels > 1,500 mIU per mL and (3) laboratory investigations of low serum β-hCG levels (<1,500 mIU per mL) [3]. The exclusion criteria were patients who had any malignancy or had intrauterine pregnancy (non-ectopic).

The sample size of the present study was based on the study by Verma et al., where the most common site of ectopic pregnancy was the fallopian tube as seen in 24 (85.7%) patients [2]. Taking the study values as a reference with the alpha error of 0.05 and power of the study as 80% and the margin of error as 10-15%, the sample size of the present study was calculated to be 75.

Written informed consent was acquired from all patients. Before starting the study, Institutional Ethical clearance was obtained (IEC, Rajendra Institute of Medical Sciences, Ranchi, Jharkhand; 391 IEC RIMS, dated 03.12.2021). The details of the patients regarding age, socioeconomic status (as per Kuppuswamy Socioeconomic Status Scale), referral, symptoms, and obstetric history were recorded in the study proforma. Signs and symptoms of patients at the time of presentation were recorded. Ultrasound (USG) was done, and the site of ectopic pregnancy was determined. Management of patients was done according to hospital protocol; outcomes were recorded.

The outcome measures were the incidence of ruptured ectopic, the need of blood transfusion in the ruptured cases and mortality. The patients were monitored and the details were recorded till the time of discharge from the hospital or death. No further follow-up of the patients was done after discharge.

Statistical analysis

The data were presented as number and percentage and mean±SD and median 25th to 75th percentile. Tables and graphs were created using the data that was entered into a Microsoft Excel sheet (Microsoft® Corp., Redmond, WA, USA). The Statistical Package for the Social Sciences (SPSS) software (IBM Corp., Armonk, NY, USA), version 25.0, was used for the analysis. p<0.05 was considered statistically significant.

## Results

Out of a total of 1350 women visiting the OPD during the study period, EP was seen in 75 women (frequency: 75/1350=5.5%). The mean (SD) age of the patients was 29 (3.8) years. The majority of the patients [43 (57%)] belonged to the lower middle class followed by the upper middle class [22 (29%)], while five (7%) cases each belonged to the poor section and high section. The majority [47 (63%)] were referral cases and 28 (37%) cases came directly. Out of 75 cases of EPs, the classical triad was present in 28 (37.3%) cases. Amenorrhea was the most common symptom seen in 73 (97.33%) cases followed by pain in the lower abdomen in 71 (94.67%) cases, bleeding per vagina (PV) in 30 (40%) cases, and syncope in six (8.00%) cases. The majority of patients were multigravida (i.e. >G4) [21 (31%)] cases, followed by Gravida-3 [18 (27%)] whereas the frequency of Gravida-2 and primigravida was 14 (21%) cases each (Table 1).

**Table 1:** Baseline clinical and demographic characteristics The data has been represented as N, % and mean±SD.

Parameter	Mean±SD	Number	Percentage
Mean age	29+-3.8 y		
<20 year		0	0%
20-30 years		45	60%
>30 years		30	40%
Socioeconomic status			
Poor Section		5	7.00%
Lower Middle		43	57.00%
Upper Middle		22	29.00%
High Section		5	7.00%
Referral		47	63.00%
In House		28	37.00%
Symptoms of Ectopic Pregnancy			
Pain in Abdomen		71	94.67%
Bleeding		30	40.00%
Syncope		6	8.00%
Amenorrhea		73	97.33%
1 month		3	4.00%
2 months		31	41.33%
3 months		7	9.33%
4 months		1	1.33%
11/2 months		22	29.33%
21/2 months		9	12.00%
Obstetric history			
Primigravida		14	21.00%
G2		14	21.00%
G3		18	27.00%
>G4		21	31.00%

Out of 75 patients, right-sided tube involvement was seen in 36 (48%), left-sided tube involvement in 38 (51%), and one (1%) had involvement of both tubes.

Ultrasound reporting was most helpful in doubtful cases and in expediting the management, which resulted in better outcomes. Most of the cases [65 (86.67%)] came with a USG report whereas 10 (13.33%) patients came without a USG report, who underwent USG in the hospital. Fourteen cases (18.67%) came with report suggestive of rupture ectopic, 15 (20%) cases with collection in pouch of Douglas (CPOD), 25 (33.33%) cases had tubo-ovarian mass (TOM) with gestational sac, six (8%) cases had tubo-ovarian mass with gestational sac with cardiac activity (TOM1), three (4%) cases had tubo-ovarian mass with gestational sac without cardiac activity (TOM2), one (1.33%) patient had USG s/o bilateral tubo-ovarian mass and one patient with broad ligament.

In the majority of patients [28 (37.33%)], the ampullary region in the fallopian tube was the site of EP, followed by fimbrial in 14 (18.65%) cases, cornual in 10 (13.33%) cases, ovarian in nine (12%) cases, isthmus in seven (9.33%) cases, infundibulum in five (6.67%) cases, broad ligament ectopic in one (1.33%) case whereas one patient had negative finding.

The most common risk factor was PID in 26 (19.5%) cases followed by a history of medical termination of pregnancy (MTP) in 20 (15%) cases, previous cesarean section in 19 (14.5%) cases and a history of spontaneous abortion in 15 (11.25%) cases. The risk factors are shown in Table 2.

**Table 2:** Risk factors of ectopic pregnancy BL: bilateral; H/O: history of; IUCD: intrauterine contraceptive device; LSCS: lower segment cesarean section; MTP: medical termination of pregnancy; OCP: oral contraceptive pill; PID: pelvic inflammatory disease; TB: tuberculosis The data has been represented as N, %

Risk Factor of Ectopic Pregnancy	No. of Cases	Percentage (%)
H/O PID	26	19.5
H/O TB	7	5.25
H/O MTP	20	15
H/O Spontaneous Abortion	15	11.25
H/O Infertility	10	7.5
LSCS	19	14.25
Appendicectomy	03	2.25
BL Tubectomy	03	2.25
Ovulation induction	04	3
IUCD	07	5.25
OCP PILLS	02	1.5

On per vaginal examination, cervical motion tenderness was seen in 59 (78.67%) cases, fullness in fornices in 57 (76%) cases, adnexal mass in 36 (48%) cases and size of the uterus was bulky in 51 (68%) cases and was normal in size in 24 (47.05%) cases.

Surgical management was done in 74 (98.67%) cases and one (1.33%) case underwent medical management (methotrexate). In surgical management, salpingectomy (S) alone was done in 47 (62.67%) cases whereas salpingectomy + contralateral tubal ligation (S+C/TL) was done in 17 (22.66%) cases, salpingo-oophorectomy (SO) alone done in two (2.67%) cases, salpingo-oophorectomy + contralateral tubal ligation (SO+C/TL) was done in three (4%) cases, one (1.33%) case underwent oophorectomy (OP) alone whereas in one (1.33%) patient oophorectomy+contralateral ligation (OP+C/TL) was done. Bilateral ectopic pregnancy was present in one (1.33%) case in which bilateral salpingectomy (BLS) was done and one (1.33%) patient underwent salpingectomy + horn resection (HR+S). Contralateral tubal ligation was done in most of the multiparous women after proper consent as part of sterilization.

Out of 74 cases operated, 18 (24%) cases had specific intraoperative findings. Adhesions were present in 10 (13.3%) cases, tubercles over the fallopian tube in four (5.5%) cases, and central line insertion and contralateral adnexa were unhealthy in one (1.3%) case.

Out of 75 patients, rupture EP was present in 71 (95%) cases. Out of 71 (95%) ruptured cases, hemoperitoneum was present in all cases; 500 ml hemoperitoneum was seen in 27 (36%) cases, <500 ml was seen in two (2.67%) cases whereas >500 ml hemoperitoneum was present in 46 (61.33%) cases. Blood transfusion was needed in 71 (94.67%) cases. In 24 (32%) cases, 1 unit PRBC was transfused, in 27 (36%) cases 2 unit PRBC, in 14 (19%) cases 3 unit PRBC, in five (7%) cases 4 unit PRBC, and in one (1%) patient >4 unit PRBC was transfused. Wound gape was present in eight (11%) cases while one (1%) patient needed ventilatory support/ICU admission and had mortality. One patient (1%) had anti-tuberculosis therapy (ATT)-associated complication (Table 3).

**Table 3:** Postoperative complications The data has been represented as N, %. ATT: anti-tuberculosis therapy; ICU: intensive care unit

Complications	N	%
Ruptured	71	95%
Hemoperitoneum	71	95%
<500 ml	2	2.67%
500 ml	27	36.00%
>500 ml	46	61.33%
Wound gape	8	11.00%
*Anti*-*tuberculosis therapy* (ATT)-associated complication	1	1.00%
Ventilatory support/ICU admission	1	1.00%
Blood transfusion	71	94.67%
1 unit PRBC	24	32.00%
2 unit PRBC	27	36.00%
3 unit PRBC	14	19.00%
4 unit PRBC	5	7.00%
5 unit PRBC	1	1.00%

## Discussion

Rising rates of sexually transmitted diseases (STDs), induced abortions, changes in lifestyle and social life, childbearing at a late age, assisted reproductive technologies, and improvements in diagnostic methods are all factors that contribute to an increase in EP around the world [7].

The present study reports a frequency of 75 patients with ectopic pregnancy (5.5%). In comparison, in an Indian study by Prasanna et al. [7], the frequency of EP was 1.8%, while another Indian study by Nethra et al. [6] reports a frequency of 1.38%. Verma et al. [2] reported frequency of EP to be 2.3%. A higher incidence of EP was noted by Kalyankar et al. [5], which was 3.95 per 1,000 pregnancies. Our study reported the EP on the higher side which might be attributed to more referrals and lower socio-economic status of the population catered.

Ectopic pregnancies occur at any age. Most of the patients (60%) in the present study belonged to 20-30 years of age, of the lower middle class (57%), were referred cases (63%), and multigravida (79%). The classical triad of EP (triad of amenorrhoea, followed by vaginal bleeding and pelvic pain) was present in 28 (37.3%) cases. The most common symptom was amenorrhea (97.33%) followed by pain in the lower abdomen (94.67%) and bleeding per vagina (40%). The findings were similar to that of Verma et al. [2], as most of the women were 21-30 years old (46.4%), and were primipara (62.1%). The classical triad of EP was seen in 39% of patients. The most common symptom was amenorrhea (93%), followed by abdominal pain (82%) and vaginal bleeding (51%). Kalyankar et al. [5] found that the classical triad of EP was seen in 45.38% of cases. The most common symptom was pain in the abdomen (90.76%), amenorrhea in 79.23% of cases, and bleeding PV in 63.07% of cases. Nethra et al. [6] reported that the majority of women were 21-30 years of age (63%), were multigravida (76%), and had lower socioeconomic status (74%). Amenorrhea, pain in the abdomen, bleeding per vagina, and fainting and syncopal attack were present in 96%, 88%, 78%, and 16% cases, respectively.

The similarity in the age group is due to the fact that in India, the majority of women get married young and start families [8]. At this age, sexual and reproductive activities are at their peak. Another most probable explanation for the peak incidence between the age group 20-30 years is that the risk of repeated PID is more which may induce tubal damage and also due to anatomic and functional age-related changes in the fallopian tube due to these factors women at this age group are more prone to EPs [9-15]. Low socioeconomic status women lack immunity and practice poor personal cleanliness, making them more susceptible to pelvic inflammatory illnesses like TB. Multiple pregnancies and infections that cause tubal damage are likely to be the causes of the more occurrence of EP in multigravida [7, 16].

The most common risk factor in the present study was found to be a history of PID that was seen in 26 (19.5%) cases, followed by h/o MTP (15%), previous cesarean section (14.5%), h/o of spontaneous abortion (11.25%), h/o infertility (7.5%), whereas seven patients (5.25%) conceived after removal of intrauterine contraceptive devices (IUCDs), four (3%) cases underwent ovulation induction, three patients conceived due to ligation failure, other than pelvic surgeries, three patients had h/o appendicectomy, and two patients had h/o oral contraceptive pill (OCP) intake. This was supported by the findings of previously conducted studies.

Verma et al. [2] reported that one or two risk factors of EP were present in 64.2% of the patients where most of the patients (30.2%) had PID and a history of induced abortion (27.3%). Other risk factors are a history of tubectomy (1.8%), previous laparotomy (1.4%), history of infertility treatment (1.2%), previous ectopic (1.4%), and ATT intake (0.9%). The study by Kalyankar et al. [5] also observed PID to be the most common risk factor (17.69%), with other factors being infertility (10.76%) abortion and dilation and curettage (D&C) (12.30%), previous ectopic (6.15%), sterilization (17.69%) and IUCD use (2.30%). Nethra et al. [6] observed that a history of PID was present in 28% of the patients. History of prior abortion was seen in 16% of cases.

Similarly, in a study conducted by Gothwal and Pathak [17], the most common risk factor was found to be PID as seen in 45% of cases, with other risk factors being abortions, tubal sterilization and Caesarean section in 15% of cases each, respectively; intra uterine contraceptives in 10% of cases; 2 (5%) women conceived following infertility treatment, tubal abortion in 5%; and history of appendectomy, ovariotomy and ovarian cystectomy in 7.5%, 5%, and 5% cases, respectively.

PID and repeated abortions remain a major cause of EP on account of the causation of scar tissue in the fallopian tube that may arise in the event of trauma or untreated long-standing PID [9]. Endosalpingitis can cause ectopic implantation by damaging the mucosa and entrapping the migrating embryo. Exosalpingitis causes peritubal adhesions, which hinder peristaltic motion and result in insufficient transport. The post-abortive infections that result in tubal damage are what explain the association between previous abortions and EP. These post-abortive infections result from illegal abortions not performed using aseptic procedures and inadequate antibiotic treatment. Previous ectopic pregnancies reflect the underlying tubal disease, which is frequently bilateral, increasing the chance of an ectopic pregnancy. EP can occur due to poor surgical technique and the development of peritubal fistulas. Edematous, congested, and friable tubes in the postpartum period enhance the risk of incomplete tubal occlusion leading to EP [6,7].

In the present study, the most common site of EP was found to be ampullary in 28 (37.33%) cases and the rarest site was broad ligament in one (1.33%) case. Verma et al. [2] reported that out of 28 EPs, 24 were tubal ectopic and four were ovarian EP. Kalyankar et al. [5] also found fallopian tubes to be the most common site of EP (83.9%), with 72.73% being in the ampulla. Other sites were ovary (6.78%) and cornua (5.08%). Andola et al. [18] found that the most common site was ampullary (61.90%), followed by fimbrial (11.90%), isthmal (9.52%), ampullary + isthmal (7.14%), cornual (4.76%), cervical (2.38%), and ovary (2.38%). Ampulla of the fallopian tube remains the commonest implantation site in cases of EP on account of being the most distensible and thick portion in the whole fallopian tube - allowing for a better space for implantation similar to the uterus [12].

In the present study, all except one patient underwent surgical management whereas one case received medical management by giving methotrexate. The most common surgical procedure was salpingectomy (S) alone (62.67%) followed by salpingectomy + contralateral tubal ligation (S+C/TL) in 22.66% of cases. In comparison, in the study by Andola et al. [18], the main modality of treatment was surgery (88.09%) and the secondary modality was medical management (7.1%). Laparoscopic unilateral salpingectomy was the most common surgery (35%) and unilateral salpingectomy was done in 15% of cases and laparoscopic salpingostomy in 10% of cases.

In Wakankar and Kedar's [19] study, out of 52 cases, 51 underwent surgical management and one case (1.9%) received medical management. Partial salpingectomy was done in most of the patients (65.38%) followed by complete salpingectomy (19.23%). Ranji et al. [3] noted that expectant management and medical therapy were offered in 15.9% and 29.4%, respectively. Surgery was done in 47.9% of cases. Salpingectomy (S) alone was done in 78.1 cases and laparoscopy in 15.1% of cases.

In the present study, 71 cases (95%) were ruptured cases of EP. Hemoperitoneum was present in all ruptured cases requiring blood transfusion due to bleeding. Eight patients (11%) had wound gape and ATT-associated complication was present in one patient. One patient needed ventilatory support/ICU admission and had mortality. In comparison, Ranji et al. [3] found that 46.2% of the cases were ruptured out of 119 cases of EP. Blood transfusion was needed in 27.7% of patients. Wakankar and Kedar [19] in their study found that 86.61% of cases were of ruptured EPs. Hemoperitoneum less than 500 ml was present in 21.15% of cases, and above 500 ml in 63.46% of cases. Postoperative complications were hospital stay >10 days in 21.15% of cases, wound complication in 9.61 of cases, ICU admission in 13.46% of cases, and general anaesthesia was needed in 51.92% of cases. The mortality rate was 0%. Andola et al. [18] observed in their study that blood transfusion was done in 47.62% of the cases either pre-, intra- or postoperatively. Ahirwar et al. [20] reported that ruptured ectopic pregnancy was present in 79.3% of cases. Hemoperitoneum of more than 500 ml was present in 43.6% of cases, and 39.3% had hemoperitoneum <500 ml. ICU admission was needed in 46.5% of patients. Hospital stay duration was 6-10 days in 65% of cases. Blood transfusion was needed in 93.6% of patients. The rate of maternal morbidity was 1.4% and no mortality occurred.

The findings of our study and other studies show that the majority of EP present as ruptured which may be due to late presentations (increased gestational age), less awareness, and less health-seeking behaviour among the patients [15].

Limitations

This study was a single-centered study, thus its results are not applicable to all populations. This study was conducted in a setting that caters to patients mainly from lower or middle socioeconomic status and thus data mainly reflects the situation in this cohort. Moreover, there were few cases who received medical management.

## Conclusions

To conclude, the frequency of EP was 5.5%. The women in the maximum reproductive age group, that is, 20-30 years, multigravida, and those from lower socioeconomic class were most affected. The most common symptoms were amenorrhea and pain in the lower abdomen. Ampullary and fimbrial was the site of EP in the majority of patients. The history of PID and MTP were the most common risk factors. The complications were rupture ectopic and hemoperitoneum needing blood transfusion with a single mortality.

The results led to the conclusion that ectopic pregnancy-related morbidity and death can be decreased by prompt identification and treatment in early pregnancy units using point-of-care USG. Moreover, a proper evaluation of pregnancy with associated risk factors is mandated to rule out ectopic cases of pregnancy.
